# Genetic characterization of an H3N2 canine influenza virus strain in China in 2023—acquisition of novel human-like amino acid substitutions

**DOI:** 10.3389/fvets.2025.1552115

**Published:** 2025-03-03

**Authors:** Sihan Li, Liangyu Chu, Yancheng Zhang, Yaxuan Yu, Guoqing Wang

**Affiliations:** ^1^College of Medicine and Biological Information Engineering, Northeastern University, Shenyang, China; ^2^College of Life and Health Sciences, Northeastern University, Shenyang, China

**Keywords:** H3N2 canine influenza virus, dog, HA1 202I, M1 227T, PB2 107 N

## Abstract

Around 2005, influenza A virus (IAV) once again crossed species barriers and established a stable lineage within canine populations. Currently, avian-origin H3N2 canine influenza virus (CIV) is the only strain of influenza that is stably circulating in canine populations. Initially, this virus was detected exclusively in Asian countries, such as China and South Korea. However, in 2015, it was isolated from dogs in the United States, where it subsequently caused a large-scale outbreak. Since its initial isolation, the H3N2 CIV has demonstrated the ability to infect mammals, including cats. Throughout its spread, the virus has continuously enhanced its adaptability to mammalian hosts, posing a potential public health threat. To monitor the prevalence of H3N2 CIV in China, we collected 131 nasal swabs from dogs and cats with respiratory symptoms between December 2023 and February 2024 in Beijing, Changchun, Shenyang, Hohhot, and Yunfu. From these samples, one influenza virus strain was successfully isolated. Through whole-genome sequencing and phylogenetic analysis, this strain was identified as avian-origin H3N2 CIV. All eight gene segments exhibited amino acid substitutions, with PB2 107 N, HA1 202I, and M1 227T positions being identical to those found in the currently circulating H3N2 human influenza virus (HuIV). Interestingly, by around 2021, the H3N2 HuIV had already completed the PB2 107 N substitution. Our study indicates that H3N2 CIV is evolving toward increased adaptability to mammalian hosts, highlighting the necessity for strengthened monitoring and risk assessment.

## 1 Introduction

Influenza A virus (IAV), a member of the *Orthomyxoviridae* family, is an enveloped virus with eight single-stranded, negative-sense RNA genomic segments (1). The natural reservoirs of IAV are believed to be migratory waterfowl. Through antigenic drift and antigenic shift, IAV has achieved infection across multiple mammalian species and established stable lineages in humans, swine, horses, and dogs. To date, IAV has been classified into 18 HA and 11 NA subtypes (2). Recently, a novel HA subtype, H19, was detected in *Aythya ferina* (3).

Canine influenza is an acute, contagious respiratory disease in dogs caused by the canine influenza virus (CIV). It can lead to symptoms such as fever, coughing, sneezing, and in severe cases, can even result in death. In 2004, the United States reported the first cases of dogs infected with equine-origin H3N8 canine influenza virus (4), leading to localized outbreaks. The first avian-origin H3N2 canine influenza virus was isolated from a dog nasal swab in 2006 (5). Subsequently, the virus established a stable lineage within canine populations across Asia. In early 2015, H3N2 CIV was introduced to the United States, where it rapidly spread through various animal shelters and kennels (6). Since 2016, there have been no further reports of positive cases of equine-origin H3N8 CIV (7). Currently, avian-origin H3N2 CIV is the only IAV that remains stably circulating in canine populations.

Amino acid substitutions may impact the biological characteristics of IAV within host cells. The interaction of the HA protein with sialic acid receptors is the initial step for IAV to invade host cells, where HA Q226L and G228S enhance binding affinity to human-like *α*-2,6 sialic acid receptors rather than to avian-like α-2,3 sialic acid receptors (8). PB2 E627K is considered one of the most significant markers for mammalian adaptation, effectively enhancing the polymerase activity of avian influenza virus (AvIV) in mammalian cells and its replication capability at 33°C (9).

Due to the influenza virus’s propensity for reassortment and the increasing adaptability of H3N2 CIV to mammalian hosts during its ongoing circulation (10), concerns about its public health risk have intensified. Due to the presence of both *α*-2,3 and α-2,6 sialic acid receptors on porcine respiratory epithelial cells, pigs can be infected by both AvIV and human influenza virus (HuIV), making them a potential intermediate species for influenza virus reassortment (11). Similarly, canine respiratory epithelial cells also contain both types of sialic acid receptors (12). Dogs have been reported to be infected with highly pathogenic H5N1 AvIV and pandemic H1N1 (pdmH1N1) (13, 14). Additionally, recombinant strains originating from different hosts have also been isolated in dogs (15–17). Cats have shown susceptibility to early isolates of H3N2 CIV (18), and they may potentially serve as intermediate hosts for the transmission of AvIV to humans (19, 20).

Between December 2022 and February 2024, we collected 431 nasal swabs from dogs with respiratory symptoms across multiple provinces in China. From these samples, we successfully isolated an avian-origin H3N2 CIV. This virus demonstrates a trend toward increased adaptability to mammalian hosts, exhibiting novel amino acid substitutions in all eight gene segments and acquiring substitutions consistent with the currently circulating H3N2 HuIV. Notably, this includes the PB2 107 N substitution, which was completed by H3N2 HuIV between 2018 and 2023. Consequently, IAV carried by companion animals have an increasing potential to cause public health issues, highlighting the need for enhanced monitoring and risk assessment of H3N2 CIV.

## 2 Materials and methods

### 2.1 Samples collection and preparation

Between December 2022 and February 2024, 431 nasal swabs were collected from dogs with respiratory symptoms in Beijing, Changchun (Jilin Province), Shenyang (Liaoning Province), Hohhot (Inner Mongolia), and Yunfu (Guangdong Province), China. The swabs were stored in 1.5 mL centrifuge tubes containing 1 mL of DMEM and preserved at −80°C for future use. Each sample was collected after permission was obtained from the owner, and the procedures met the requirements of the Northeastern University Biological and Medical Ethics Committee.

### 2.2 Virus isolation, RNA extraction, and detection

Each collected nasal swab sample from dogs was filtered through a 0.45 μm membrane before being inoculated into 9-day-old SPF chicken embryos. After 48 h, the allantoic fluid from the embryos was harvested and subjected to a hemagglutination (HA) test (21). HA-positive samples were stored at −80°C. Viral RNA was extracted from 250 μL of HA-positive allantoic fluid or nasal swab samples using the RaPure Viral DNA/RNA Kit (Magen, China) according to the manufacturer’s instructions. cDNA synthesis was performed on 7 μL of the extracted RNA using the HiScript II 1st Strand cDNA Synthesis Kit (Vazyme, China) with the Uni12 primer (5’-AGCAAAAGCAGG-3′) (22). The cDNA samples were then amplified by PCR using Phanta Flash Super-Fidelity DNA Polymerase (Vazyme, China). PCR detection was performed on HA-positive allantoic fluid samples using primers (IAV-M F/R) designed to target the M gene of IAV. Upon confirmation of IAV nucleic acid positivity, the full genome of IAV was amplified from the nasal swab sample. The PCR products were analyzed by nucleic acid electrophoresis on a 1% agarose gel, and DNA fragments of the expected size were purified. Details of the primers are provided in Table 1.

**Table 1:** Primers used for virus detection and genome sequencing.

Primer	Primer sequence (5′ to 3′)	
	F	R
PB2	gacctccgaagttgggggggAGCGAAAGCAGGTC	ttttgggccgccgggttattAGTAGAAACAAGGTCGTTT
PB1	gacctccgaagttgggggggAGCGAAAGCAGGCA	ttttgggccgccgggttattAGTAGAAACAAGGCATTT
PA	gacctccgaagttgggggggAGCGAAAGCAGGTAC	ttttgggccgccgggttattAGTAGAAACAAGGTACTT
HA	gacctccgaagttgggggggAGCAAAAGCAGGGG	ttttgggccgccgggttattAGTAGAAACAAGGGTGTTTT
NP	gacctccgaagttgggggggAGCAAAAGCAGGGTA	ttttgggccgccgggttattAGTAGAAACAAGGGTATTTTT
NA	gacctccgaagttgggggggAGCAAAAGCAGGAGT	ttttgggccgccgggttattAGTAGAAACAAGGAGTTTTTT
M	gacctccgaagttgggggggAGCAAAAGCAGGTAG	ttttgggccgccgggttattAGTAGAAACAAGGTAGTTTTT
NS	gacctccgaagttgggggggAGCAAAAGCAGGGTG	ttttgggccgccgggttattAGTAGAAACAAGGGTGTTTT
IAV-M	CAGAGACTKGARGATGTNTTTGC	CTACGCTGCAGTCCTCGCTC

F, forward primer. R, reverse primer. The lowercase letters denote the homologous sequences of the pHW2000 vector.

### 2.3 Plasmid construction and whole genome sequencing

The purified IAV gene fragments were seamlessly cloned into the pHW2000 vector using the 2 × MultiF Seamless Assembly Mix (Abclonal, China). The plasmids were then transformed into *Escherichia coli* DH5α competent cells (ToloBio, China) and cultured on LB agar plates containing ampicillin for 12 h. Bacterial suspensions containing positive clones were identified and subsequently subjected to sequencing (Sangon, China).

### 2.4 Sequence analysis and phylogenetic analyses

Nucleotide similarity analysis of the eight gene segments of IAV was performed using the BLAST tool from the NCBI database.^1^ Sequences for H3N2 AvIV, H3N2 CIV, H3N2 swine influenza virus (SIV), H3N8 equine influenza virus (EIV) and H3N2 HuIV were downloaded from NCBI. We generated single-codon alignments for each segment using MAFFT version 7.52: PB2 (2,280 nt), PB1 (2,274 nt), PA (2,151 nt), HA (1,698 nt), NP (1,497 nt), NA (1,413 nt), M (982 nt), and NS (838 nt). A subset of H3N2 CIV sequences was selected based on the year and geographic origin. Phylogenetic trees for the eight viral gene segments were constructed using IQ-TREE version 2.3.6, based on the coding sequence (CDS) nucleotide sequences of the viral genes. The tree construction employed the Maximum Likelihood (ML) method with 1,000 ultrafast bootstrap replicates (23). To ensure the accuracy of the phylogenetic analysis, the optimal substitution model was applied to each gene segment. Specifically, the GTR + F + G4 model was used for the HA, PB2, and NP gene segments, the TVM + F + I + G4 model for the NA gene segment, the TPM2u + F + G4 model for the PB1 gene segment, the TVM + F + I model for the PA gene segment, the TVMe+G4 model for the M gene segment, and the TVM + F + G4 model for the NS gene segment. Amino acid distribution analysis was conducted based on the new substitution sites identified in the H3N2 CIV isolates, comparing H3N2 AvIV, H3N2 CIV, and H3N2 HuIV. All sequences for H3N2 AvIV, H3N2 CIV, and H3N2 HuIV were sourced from the NCBI database, including 3,052 H3N2 CIV sequences, 176,393 H3N2 HuIV sequences, and 4,356 H3N2 AvIV sequences.

## 3 Results

### 3.1 Virus detection and isolation

**Figure 1:**
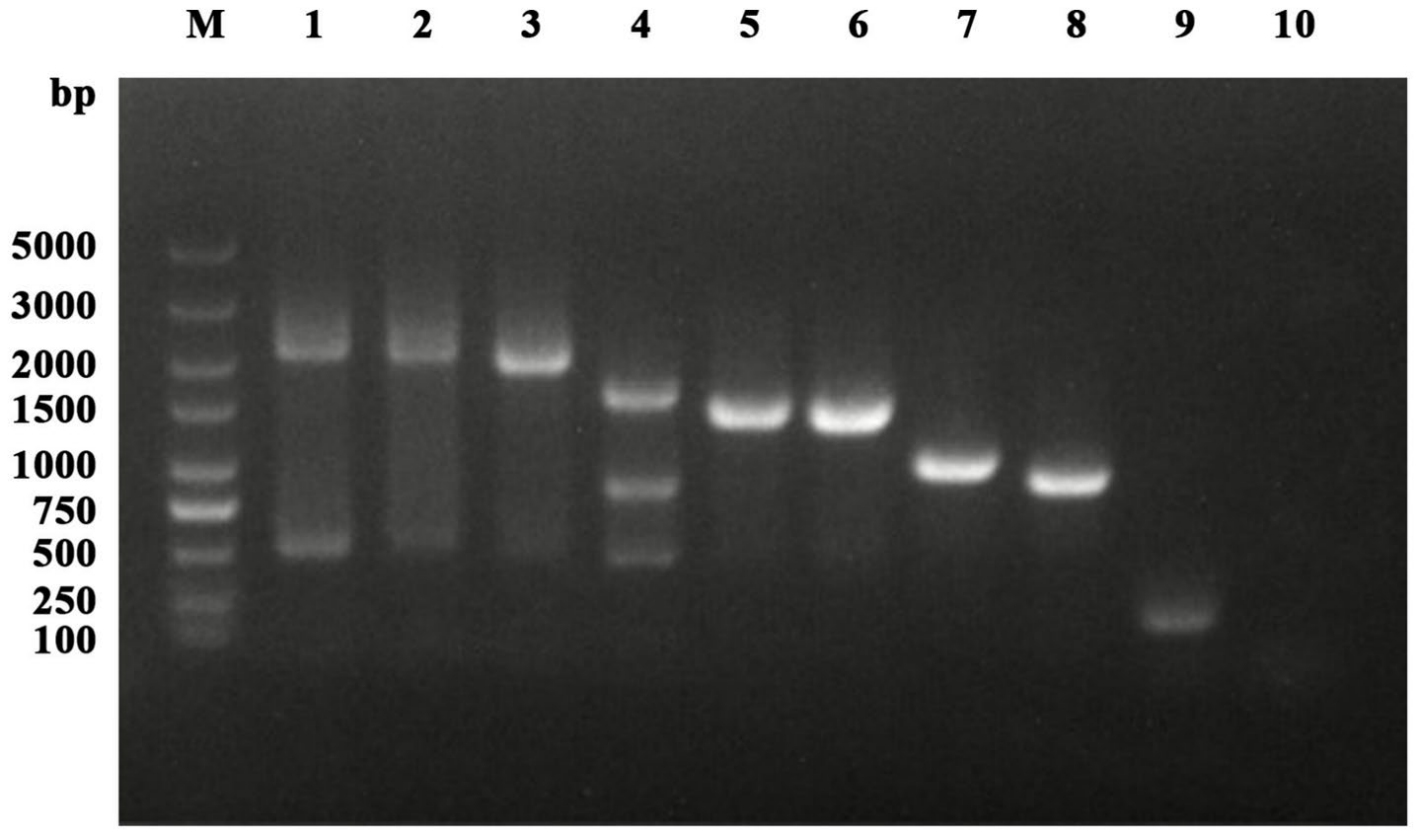
Detection and amplification results of IAV. Lanes 1 to 8 represent PB2, PB1, PA, HA, NP, NA, M, and NS, respectively. Lanes 9 and 10 are the positive and negative controls for IAV-M detection.

Among the 431 nasal swab samples, HA positivity was observed in only one sample following inoculation in chicken embryos. A strain of influenza virus was isolated from a nasal swab sample of a dog collected in Changchun, Jilin Province. The dog, a 3-month-old male, presented with cough, nasal discharge, and fever on December 28, 2023. The PCR results for the nasal swab sample were positive for IAV nucleic acid and all eight gene segments of the IAV were successfully amplified in full sequence (Figure 1).

### 3.2 Molecular characteristics of the H3N2 CIV

To study the genetic characteristics of this IAV strain, we amplified the eight gene segments of the virus and performed nucleotide similarity analysis on the viral genomic coding sequences (CDS). The results, as shown in Table 2, demonstrate that the nucleotide similarity of the eight gene segments to recent H3N2 CIV strains circulating in China and the United States is no less than 99.16%. Therefore, the virus is identified as an avian-origin H3N2 CIV, and it has been designated as A/canine/ChangChun/01/2023 (CIV-CC23). The sequence of CIV-CC23 has been deposited in the GenBank database under accession numbers PQ374162 to PQ374169.

**Table 2:** Nucleotide similarity analysis of H3N2 CIV.

Segment	Strain name	Accession number	Similarity
PB2	A/canine/Florida/CAIS-F1/2021	OR732186.1	99.61%
PB1	A/canine/Florida/CAIS-H1/2021	OR732195.1	99.30%
PA	A/canine/Texas/042018–3/2023	OR738675.1	99.35%
HA	A/canine/Florida/CAIS-C2/2021	OP364488.1	99.24%
NP	A/canine/Texas/21–011409-001/2021	MZ441118.1	99.47%
NA	A/canine/Alabama/194456–1/2022	OR738646.1	99.50%
M	A/canine/California/M21-32151-10-1/2021	OR732120.1	99.29%
NS	A/canine/Beijing/18/2019	ON877623.1	99.16%

Sequence analysis of CIV-CC23 compared to the strains with the highest nucleotide similarity revealed that CIV-CC23 has acquired amino acid substitutions in all eight gene segments (Table 3). When incorporating all previously isolated H3N2 CIV amino acid sequences, we found that half (13/26) of these amino acid substitutions are entirely novel and have not been observed in H3N2 CIV before. Notably, the PA protein has acquired the highest number of novel substitutions, totaling 3.

**Table 3:** Differences in amino acid distribution between H3N2 AvIV, CIV, and HuIV.

Segment	Position		H3N2 AvIV residue(s) (%)	H3N2 CIV residue (s) (%)	CIV-CC23	H3N2 HuIV residues(s) (%)
PB2	107		S (98.81) N (1.19)	S (100.00)	**N**	N (49.52) D (28.94) S (21.46)
	615		I (100.00)	I (99.74) T (0.26)	T	I (99.82)T (0.02)
PB1	191		V (98.41) I (1.39)	V (100.00)	I	V (99.69) I (0.30)
	611		L (100.00)	L (99.45) M (0.55)	M	L (100.00)
	645		V (100.00)	V (93.17) I (6.83)	I	V (99.86) I (0.14)
	669		K (100.00)	K (100.00)	R	K (99.98) R (0.02)
PA	127		V (99.61) I (0.39)	V (100.00)	I	V (99.87)
	348		I (77.93) L (21.67)	I (99.21) V (0.53) S (0.26)	T	I (99.62)
	401		R (96.44) K (3.36)	K (64.64) R (35.36)	N	R (99.98)
	683		L (99.59) I (0.41)	L (94.01) *F* (5.99)	L	L (100.00)
HA	HA1	12	T (98.80) K (0.34)	T (100.00)	K	T (99.69)
		202	V (99.01) I (0.66)	V (99.49) I (0.51)	**I**	I (99.87)
		208	R (96.67) K (2.16)	R (95.14) G (4.35) K (0.51)	G	R (99.70)
		297	V (99.33) I (0.67)	V (98.98) I (1.02)	I	V (99.88)
	HA2	100	V (90.00) I (10.00)	V (98.98) I (1.02)	I	V (99.93)
		147	A (88.87) T (1.69)	A (98.97) T (1.03)	T	A (99.72)
		200	V (96.96) I (2.87)	V (100.00)	A	I (72.28) V (27.64)
NP	22		A (100.00)	A (99.73) T (0.26)	T	A (99.93)
NA	48		N (98.54) S (0.81)	S (91.69) N (8.05) Y (0.26)	N	N (99.91) S (0.05)
	50		V (94.00) I (4.05) T (0.32)	I (62.08) V (35.32) T (2.6)	T	V (99.81)
	283		R (97.39) Q (0.65)	Q (64.32) R (35.68)	R	R (99.85) Q (0.12)
M	M1	192	M (99.81) I (0.19)	M (100.00)	I	M (99.94) V (0.04) I (0.02)
		227	A (96.60) T (3.40)	A (100.00)	**T**	T (99.82) A (0.13)
	M2	58	G (99.43) E (0.57)	G (100.00)	D	G (91.96)S (7.98)
NS	NS1	112	A (75.97) T (18.02)	A (99.74) V (0.26)	T	E (99.56)
		113	G (99.61) S (0.39)	G (99.74) E (0.26)	R	G (100.00)

The novel amino acid substitutions acquired by CIV-CC23 are indicated by underline. The newly acquired human-like amino acid substitutions in CIV-CC23 are indicated in bold. The HA sequence was encoded as H3 after removing the signal peptide.

### 3.3 Phylogenetic analysis of the H3N2 CIV

**Figure 2:**
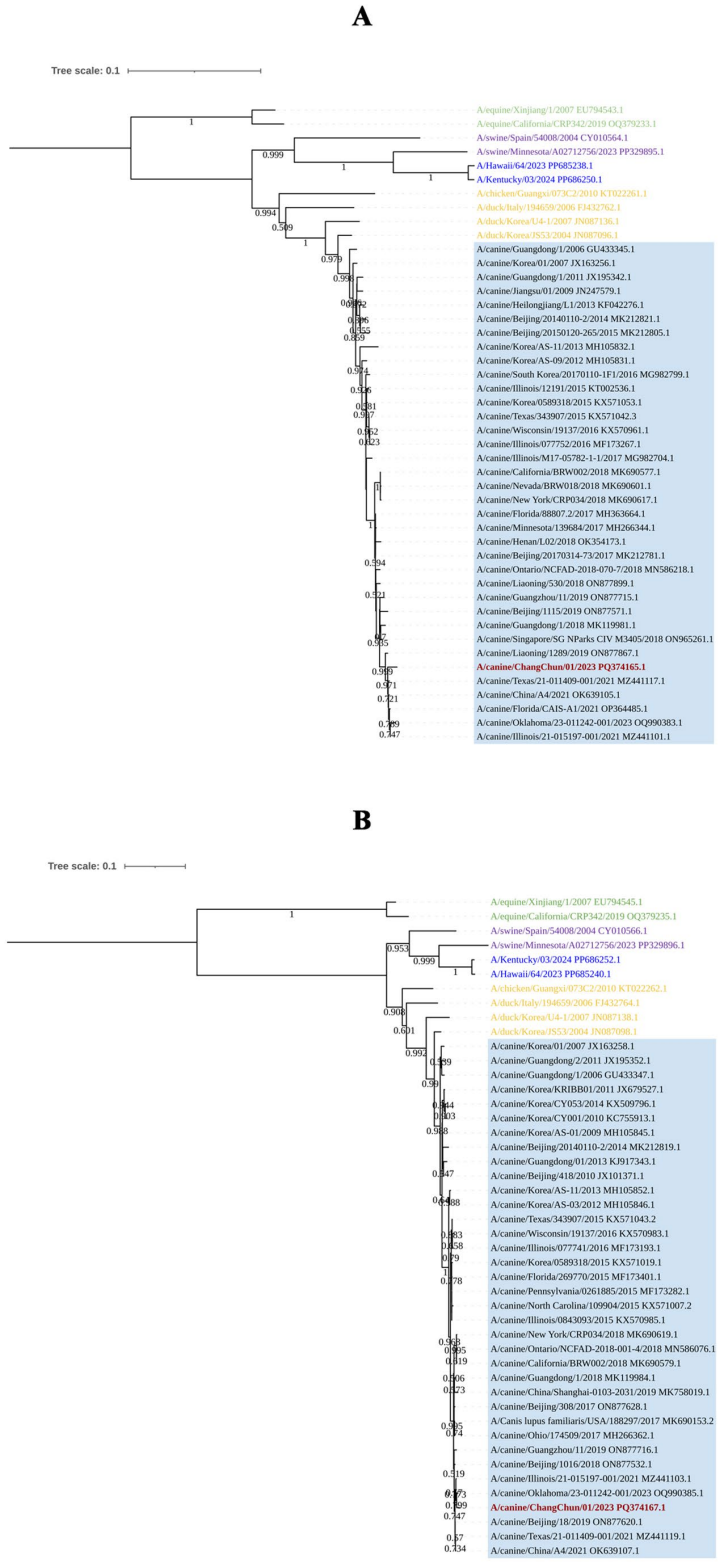
Phylogenetic analysis of the viral surface genes of CIV-CC23. **(A)** HA gene segment. **(B)** NA gene segment. The light blue squares represent avian-origin H3N2 CIV, the red branch represents CIV-CC23, the yellow branch represents H3N2 AvIV, the green branch represents H3N8 EIV, the blue branch represents HuIV H3N2, and the purple branch represents H3N2 SIV.

**Figure 3:**
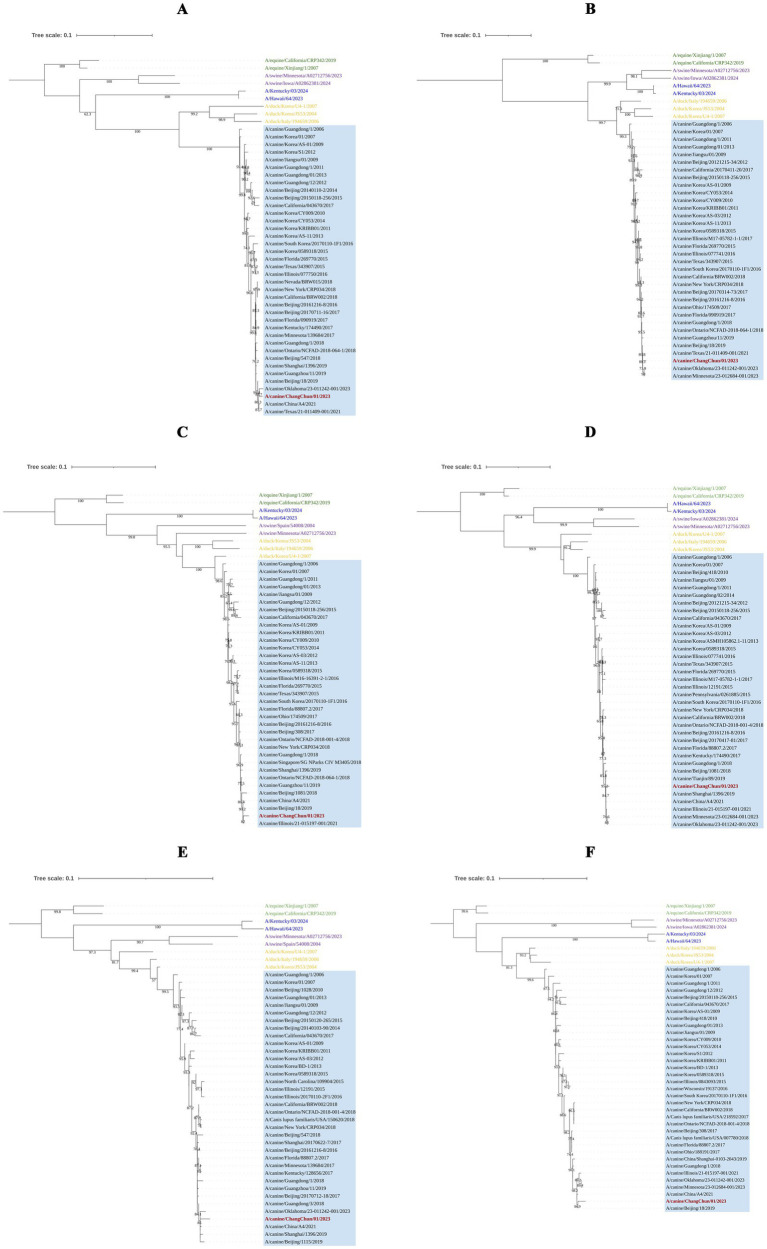
Phylogenetic analysis of the internal genes of CIV-CC23. **(A)** PB2 gene segment. **(B)** PB1 gene segment. **(C)** PA gene segment. **(D)** NP gene segment. **(E)** M gene segment. **(F)** NS gene segment. The light blue squares represent avian-origin H3N2 CIV, the red branch represents CIV-CC23, the yellow branch represents H3N2 AvIV, the green branch represents H3N8 EIV, the blue branch represents HuIV H3N2, and the purple branch represents H3N2 SIV.

To understand the origin of CIV-CC23, we conducted a phylogenetic analysis of its eight gene segments. The results, as illustrated in Figures 2, 3, indicate that CIV-CC23 is classified within the avian-origin H3N2 CIV. The HA (PQ374165) and NP (PQ374166) gene is closely related to recent isolates from both China and the United States, yet it forms a distinct small branch. The PB1 gene (PQ374163) clusters with a 2023 U.S. isolate, while the NA (PQ374167) and PB2 genes (PQ374162) cluster with A/canine/Oklahoma/23–011242-001/2023. The PA gene (PQ374164) clusters with A/canine/Illinois/21–015197-001/2021, the M gene (PQ374168) with A/canine/China/A4/2021, and the NS gene (PQ374169) with A/canine/Beijing/18/2019.

### 3.4 Acquisition of novel human-like amino acid substitutions

**Figure 4:**
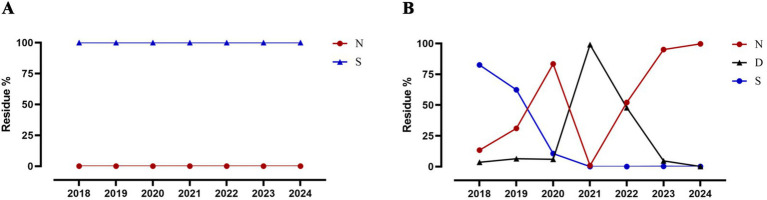
Changes in PB2 107 position in H3N2 AvIV and HuIV. **(A)** H3N2 AvIV. **(B)** H3N2 HuIV.

To investigate whether the amino acid substitutions acquired by CIV-CC23 are associated with mammalian adaptation, we analyzed the amino acid polymorphisms at these sites in H3N2 HuIV isolates from 2018 to 2024. The results, presented in Table 3, show that CIV-CC23 has acquired 3 novel human-like amino acid substitutions: PB2 107 N, HA1 202I, and M1 227T. These substitutions are prevalent in currently circulating H3N2 HuIV strains. Among them, PB2 107 N is the predominant amino acid substitution in current H3N2 HuIV strains, accounting for 99.69% (2,882/2891) of the isolates from 2024. In contrast, the predominant amino acid substitution in H3N2 HuIV in 2018 was PB2 107S, which comprised 82.58% (2,142/2594) of the isolates (Figure 4). PB2 107S is commonly found in H3N2 AvIV, comprising 98.81% (497/503) of all H3N2 AvIV isolates, and recent H3N2 AvIV isolates have consistently exhibited PB2 107S.

## 4 Discussion

Since the 20th century, IAVs have caused four pandemics and are listed by the World Health Organization (WHO) as one of the potential pathogens that could trigger the next pandemic. Each of these pandemics was attributable to host shifts of IAV and the corresponding lack of pre-existing immunity in the human population. Since its emergence in the early 21st century, avian-origin H3N2 CIV was initially confined to a few Asian countries. However, it is now reported in multiple countries worldwide, clearly indicating that H3N2 CIV has established a stable presence in canine populations. Even during the COVID-19 pandemic, despite stringent control measures, H3N2 CIV has continued to circulate among dogs in China. The ongoing adaptation of H3N2 CIV to mammals and the corresponding lack of immunity in human populations (10) underscore the importance of monitoring and addressing this virus.

In this study, we collected nasal swabs from dogs and cats with respiratory symptoms across several provinces in both northern and southern China, obtaining a total of 131 samples, including 42 from dogs and 89 from cats. We successfully isolated an H3N2 CIV from a nasal swab of a 3-month-old dog, while no influenza viruses were detected in the cat samples. Although H3N2 CIV emerged in the United States in 2015, no new variants distinct from those circulating in Asia have been reported. Compared to recent isolates from China, CIV-CC23 not only showed higher nucleotide similarity with recent U.S. isolates in nucleotide similarity analyses but also clustered within the same evolutionary branch as U.S. isolates in phylogenetic analyses. This phenomenon has also been observed in previous reports (24, 25). While we cannot rule out the possibility of imported cases, we are more inclined to believe that the decline in domestic H3N2 virus isolation rates, influenced by COVID-19-related restrictions, has led to a lack of local viral sequence information from China, especially considering that this dog is relatively young and its activity is limited to a rural area.

CIV-CC23 has acquired 3 novel human-like amino acid substitutions compared to the preceding H3N2 CIV strains. This findings suggest that H3N2 CIV evolves over time, and if it accumulates a sufficient number of human-like amino acid substitutions, it may acquire the ability to efficiently propagate in humans. Among these substitutions, PB2 107 N is an amino acid change that occurred in H3N2 HuIV around 2021. Almost all H3N2 AvIV and previous H3N2 CIV isolates have been PB2 107S, making the emergence of this substitution in CIV-CC23 noteworthy. While the precise impact of this substitution on H3N2 CIV’s adaptation to mammals remains unclear, PB2 is known to play a significant role in enhancing IAV adaptation to mammals. For example, PB2 E627K increases the virus’s polymerase activity, facilitating replication in mammalian cells (9). Similarly, PB2 S714I in H3N2 CIV has been shown to enhance RNP complex activity, virus replication, and pathogenicity in mice (26). Given our limited sample size, it remains uncertain whether PB2 107 N will be prevalent in future H3N2 CIV. This requires broader temporal and geographic monitoring to obtain a sufficient sample size. However, the relationship between mammalian adaptive changes and human-like amino acid substitutions in H3N2 CIV warrants further investigation.

Our study indicates that H3N2 CIV continues to circulate in canine populations in China, persistently acquiring human-like amino acid substitutions, which poses a potential public health risk. Therefore, a comprehensive surveillance system for H3N2 CIV should be established, with collaboration among animal hospitals, shelters, and pet trade markets nationwide. Nasal swab samples should be collected from dogs and cats presenting with respiratory symptoms to facilitate broader and more diverse studies on virus prevalence and host specificity. Additionally, it is essential to enhance monitoring and management of companion animal transportation and strengthen risk assessment for H3N2 CIV to better understand the zoonotic potential of the currently circulating strain.

## Data Availability

The datasets presented in this study can be found in online repositories. The names of the repository/repositories and accession number(s) can be found in the article/supplementary material.

## Footnotes

^1^ https://blast.ncbi.nlm.nih.gov/Blast.cgi

## References

[1] Kristensen C Jensen HE Trebbien R Webby RJ Larsen LE. Avian and human influenza a virus receptors in bovine mammary gland. Emerg Infect Dis. (2024) 30:1907–11. doi: 10.3201/eid3009.240696, PMID: 39127127 PMC11347012

[2] Wu Y Wu Y Tefsen B Shi Y Gao GF. Bat-derived influenza-like viruses H17N10 and H18N11. Trends Microbiol. (2014) 22:183–91. doi: 10.1016/j.tim.2014.01.010, PMID: 24582528 PMC7127364

[3] Fereidouni S Starick E Karamendin K Genova CD Scott SD Khan Y. Genetic characterization of a new candidate hemagglutinin subtype of influenza a viruses. Emerg Microbes Infect. (2023) 12:2225645. doi: 10.1080/22221751.2023.2225645, PMID: 37335000 PMC10308872

[4] Crawford PC Dubovi EJ Castleman WL Stephenson I Gibbs EP Chen L. Transmission of equine influenza virus to dogs. Science. (2005) 310:482–5. doi: 10.1126/science.1117950, PMID: 16186182

[5] Li S Shi Z Jiao P Zhang G Zhong Z Tian W. Avian-origin H3N2 canine influenza a viruses in southern China. Infect Genet Evol. (2010) 10:1286–8. doi: 10.1016/j.meegid.2010.08.010, PMID: 20732458 PMC2950248

[6] Voorhees IEH Dalziel BD Glaser A Dubovi EJ Murcia PR Newbury S. Multiple incursions and recurrent epidemic fade-out of H3N2 canine influenza a virus in the United States. J Virol. (2018) 92:18. doi: 10.1128/JVI.00323-18, PMID: 29875234 PMC6069211

[7] Wasik BR Rothschild E Voorhees IEH Reedy SE Murcia PR Pusterla N. Understanding the divergent evolution and epidemiology of H3N8 influenza viruses in dogs and horses. Virus Evolution. (2023) 9:vead052. doi: 10.1093/ve/vead052, PMID: 37692894 PMC10484056

[8] Matrosovich M Matrosovich T Uhlendorff J Garten W Klenk HD. Avian-virus-like receptor specificity of the hemagglutinin impedes influenza virus replication in cultures of human airway epithelium. Virology. (2007) 361:384–90. doi: 10.1016/j.virol.2006.11.030, PMID: 17207830

[9] Zhang H Li X Guo J Li L Chang C Li Y. The PB2 E627K mutation contributes to the high polymerase activity and enhanced replication of H7N9 influenza virus. J Gen Virol. (2014) 95:779–86. doi: 10.1099/vir.0.061721-0, PMID: 24394699

[10] Chen M Lyu Y Wu F Zhang Y Li H Wang R. Increased public health threat of avian-origin H3N2 influenza virus caused by its evolution in dogs. eLife. (2023) 12:83470. doi: 10.7554/eLife.83470, PMID: 37021778 PMC10147381

[11] Ito T Couceiro JN Kelm S Baum LG Krauss S Castrucci MR. Molecular basis for the generation in pigs of influenza a viruses with pandemic potential. J Virol. (1998) 72:7367–73. doi: 10.1128/JVI.72.9.7367-7373.1998, PMID: 9696833 PMC109961

[12] Ning ZY Wu XT Cheng YF Qi WB An YF Wang H. Tissue distribution of sialic acid-linked influenza virus receptors in beagle dogs. J Vet Sci. (2012) 13:219–22. doi: 10.4142/jvs.2012.13.3.219, PMID: 23000577 PMC3467395

[13] Lin D Sun S Du L Ma J Fan L Pu J. Natural and experimental infection of dogs with pandemic H1N1/2009 influenza virus. J Gen Virol. (2012) 93:119–23. doi: 10.1099/vir.0.037358-0, PMID: 21976611

[14] Songserm T Amonsin A Jam-On R Sae-Heng N Pariyothorn N Payungporn S. Fatal avian influenza A H5N1 in a dog. Emerg Infect Dis. (2006) 12:1744–7. doi: 10.3201/eid1211.060542, PMID: 17283627 PMC3372347

[15] Chen Y Trovão NS Wang G Zhao W He P Zhou H. Emergence and evolution of novel Reassortant influenza a viruses in canines in southern China. MBio. (2018) 9:9. doi: 10.1128/mBio.00909-18, PMID: 29871917 PMC5989073

[16] Lee IH Le TB Kim HS Seo SH. Isolation of a novel H3N2 influenza virus containing a gene of H9N2 avian influenza in a dog in South Korea in 2015. Virus Genes. (2016) 52:142–5. doi: 10.1007/s11262-015-1272-z, PMID: 26757941

[17] Song D Moon HJ An DJ Jeoung HY Kim H Yeom MJ. A novel reassortant canine H3N1 influenza virus between pandemic H1N1 and canine H3N2 influenza viruses in Korea. J Gen Virol. (2012) 93:551–4. doi: 10.1099/vir.0.037739-0, PMID: 22131311 PMC3352354

[18] Song DS An DJ Moon HJ Yeom MJ Jeong HY Jeong WS. Interspecies transmission of the canine influenza H3N2 virus to domestic cats in South Korea, 2010. J Gen Virol. (2011) 92:2350–5. doi: 10.1099/vir.0.033522-0, PMID: 21715595

[19] Belser JA Pulit-Penaloza JA Sun X Brock N Pappas C Creager HM. A novel a(H7N2) influenza virus isolated from a veterinarian caring for cats in a new York City animal shelter causes mild disease and transmits poorly in the ferret model. J Virol. (2017) 91:17. doi: 10.1128/JVI.00672-17, PMID: 28515300 PMC5512233

[20] Zhao J He W Lu M He H Lai A. Emergence and characterization of a novel reassortant canine influenza virus isolated from cats. Pathogens. (2021) 10:320. doi: 10.3390/pathogens10101320, PMID: 34684269 PMC8539923

[21] Killian ML. Hemagglutination assay for influenza virus. Methods Mol Biol. (2020) 2123:3–10. doi: 10.1007/978-1-0716-0346-8_1, PMID: 32170676

[22] Hoffmann E Stech J Guan Y Webster RG Perez DR. Universal primer set for the full-length amplification of all influenza a viruses. Arch Virol. (2001) 146:2275–89. doi: 10.1007/s007050170002, PMID: 11811679

[23] Minh BQ Schmidt HA Chernomor O Schrempf D Woodhams MD Von Haeseler A. IQ-TREE 2: new models and efficient methods for phylogenetic inference in the genomic era. Mol Biol Evol. (2020) 37:1530–4. doi: 10.1093/molbev/msaa015, PMID: 32011700 PMC7182206

[24] Li Y Zhang X Liu Y Feng Y Wang T Ge Y. Characterization of canine influenza virus a (H3N2) circulating in dogs in China from 2016 to 2018. Viruses. (2021) 13:279. doi: 10.3390/v13112279, PMID: 34835084 PMC8618230

[25] Ou J Zheng F Cheng J Ye SS Ye C Jia K. Isolation and genetic characterization of emerging H3N2 canine influenza virus in Guangdong Province, southern China, 2018-2021. Front Vet Sci. (2022) 9:810855. doi: 10.3389/fvets.2022.810855, PMID: 35372528 PMC8965554

[26] Li X Jia T Wang K Wang L Zhou L Li M. The PB2 I714S mutation influenced mammalian adaptation of the H3N2 canine influenza virus by interfering with nuclear import efficiency and RNP complex assembly. Emerg Microbes Infect. (2024) 13:2387439. doi: 10.1080/22221751.2024.2387439, PMID: 39139051 PMC11328605

